# Everyday life and boredom of people living with dementia in residential long-term care: a merged methods study

**DOI:** 10.1186/s12877-024-05641-7

**Published:** 2024-12-31

**Authors:** Doris Gebhard, Julia I. Frank

**Affiliations:** Department of Health and Sport Sciences, Technical University of Munich, Georg-Brauchle-Ring 62, 80992 Munich, Germany

**Keywords:** Dementia, Long-term care, Boredom, Daily routine, Person-centred care, Merged methods

## Abstract

**Background:**

Everyday life in residential long-term care is widely portrayed as boring. However, empirical evidence on this topic remains limited, particularly for the vulnerable group of people living with dementia. A better understanding of everyday life and the associated experiences of boredom could facilitate the development of practical strategies to reduce boredom in this target group. The aim of this study is therefore to analyse everyday activities, daily routines and the frequency and types of boredom in people living with dementia in residential long-term care.

**Data and methods:**

In five long-term care facilities, participants were observed for two days in 20-minute time slots (from 7 am to 7 pm) using the Maastricht Electronic Daily Live Observation Tool. Semi-structured interviews were conducted with people living with dementia about their daily routines and experiences of boredom. Qualitative content analysis based on Mayring was applied, using the five boredom types from Goetz et al. as deductive categories. Observational data was analysed descriptively and merged with qualitative data on daily routines. In all, 46 people living with dementia (average age: 84.65 ± 7.15 years, 89.13% female, average DSS: 6.50 ± 3.15) were observed at 2760 time points. Of these, 17 participants took part in the interviews.

**Results:**

The residents spend 47.5% of their day doing nothing and follow a routine that is strongly determined by communal meals. 62.5% of participants are bored, with 18.5% describing boredom as a constant/prevalent condition in their everyday lives. All five types of boredom are reflected in the interviews, with apathetic boredom being the most common.

**Conclusions:**

Although people living with dementia follow almost the same daily routine in residential long-term care, they experience everyday life very differently, ranging from no boredom, to feelings of pleasant relaxation when bored to strongly negative feelings such as hopelessness and frustration. These findings suggest that interventions to prevent or reduce boredom need to be personalised in order to effectively combat the highly individual nature of boredom. Person-centred dementia care provides a valuable intervention strategy to meet this requirement.

**Clinical trial number:**

Not applicable.

## Background

Everyday life in residential long-term care is widely portrayed as boring, even to the extent of labelling residents as ‘bored to death’ [1, 2]. In light of the COVID pandemic, public interest in the way care home residents spend their day has increased [3], while everyday life in residential long-term care has been the subject of research for several decades. More than 50 years ago, Gottesman and Norman [4] observed and criticised that care home residents spend most of their time doing little or nothing. Nearly 25 years later, Harper Ice [5] raised the question of whether daily life in residential long-term care has changed since Gottesman and Norman’s study. The author [5] came to the same conclusions as his colleagues 25 years earlier, and added that people with cognitive impairments are the group most affected by ‘doing nothing’. Although both studies are certainly not representative, they serve to illustrate that a lack of activities in long-term care facilities have been discussed as problematic for many years.

Today, again more than two decades later, daytime activities are still one of the most frequently reported unmet need of people living in long-term care facilities [6], with up to 73.1% of residents reporting not having sufficient things to do to get through the day [7]. People with dementia are still significantly more likely to have their activity needs unmet than their cognitively healthy peer residents [7, 8]. Cohen-Mansfield et al. [9] found unmet needs related to boredom/sensory deprivation in almost two-thirds of their sample of residents with dementia, and a recent ecological assessment study found that this population spent almost half of the day (44.2%) without doing anything [10]. However, the lack of activities is not the decisive factor that makes people with dementia experience life in residential long-term care facilities as boring [11, 12].

### The experience of boredom

Although there is currently no universal definition of boredom [13, 14], some identified signature markers can help to better understand what contributes to this experience. The core of the concept of state boredom is ‘the aversive experience of wanting, but being unable’ [15 p.482] to engage in *meaningful* activities [15, 16]. Thus, an activity must fulfil at least two independent characteristics to be experienced as not boring: (1) it must be perceived as *individually meaningful* and (2) the person must *be able to engage* in it [17]. Previous research has investigated both characteristics in relation to people living with dementia.

The definition and use of the term *meaningful activity* are unclear and inconsistent [18] and are interpreted differently across disciplines such as psychology, nursing, and occupational science. However, significant progress has been made in understanding the attributes that make activities meaningful for people living with dementia. Han et al. [19] synthesized studies on the perceptions of people living with dementia and highlighted the creation of a sense of connection as the core attribute of meaningful activities. This connection encompasses three dimensions: with oneself, through activities that maintain individual identity and health; with others, through activities that foster a sense of belonging within a larger social context; and with the environment, through activities that sustain a relationship with the physical world. For people living with dementia in residential long-term care, Tierney et al. [20] provided additional clarity by identifying six attributes that make activities meaningful: An activity must be enjoyable, engaging, suited to the individual, linked to the person’s identity, related to a personally relevant goal and have a social dimension.

The second characteristic of boredom, the *inability* is widely discussed in terms of attention, i.e. a person’s cognitive ability to direct and maintain focus on an activity [16]. This ability is not necessarily associated with high cognitive performance but with a match between the cognitive demands of an activity and a person’s individual cognitive resources in order to avoid under- or overstimulation [14]. Regier et al. [21] investigated which features of an activity can improve the ability of a person living with dementia to engage in it. Tailoring the activity in terms of its implementation environment (e.g. noise, lightning, number of persons involved), complexity (e.g. degree of goal-orientation, number of execution steps, repetition), and duration (e.g. 10-minute activities that may be suitable for people with severe dementia) were found to enable individuals with mild to severe dementia to engage in meaningful activities [21].

Beyond the question of whether a person finds meaning in an activity and is able to pay attention to it, several other correlates of boredom have been identified [14]. The feeling of restriction [14, 15] appears to be a particularly relevant correlate of boredom for institutionalised people with dementia, as the transition to long-term care facilities is associated with a loss of independence and agency [22]. The lack of agency relates both to individual activities and to daily life in residential long-term care. People with dementia report having not much influence on the choice of activities offered in the facility, even if they find them boring, such as quiz games or memorising proverbs [11]. In addition, daily life is described as regimented by the rules and routines of the institution and therefore ‘boring in the long run’ [12 p.9, 23]. The days are characterised as monotonous and very similar, with few opportunities to decide on the routines of everyday life [12, 23, 24]. The perceived helplessness may contribute to some kind of resignation, as some residents feel that they have lost interest in doing anything at all [11]. However, there are only very few studies that explore the everyday routines of people living with dementia in residential long-term care facilities [25]. Existing studies focus on specific periods or activities throughout the day, such as mealtimes [26, 27], or rely on case studies [28] and field notes [25]. Thus, the current state of research does not provide sufficient information about the detailed daily routines of people living with dementia in care facilities, nor about their perception of daily routines that go beyond generalised individual statements.

However, alternative perspectives on boredom emphasize its functional role, highlighting its potential to promote positive outcomes [29]. Central to this theory is the concept of boredom as a functional emotion—a signal that the current situation is unfulfilling, motivating individuals to pursue new, meaningful goals and stimulating experiences [17, 30, 31]. This view frames boredom as a driving force for productivity and action, fostering greater satisfaction and development. The potential of boredom to inspire creativity and growth has been widely studied in children and adolescents [32]. However, research on how this potential manifests in older adults, particularly those living with dementia, remains scarce. However, restricted autonomy and lack of opportunities for action in the daily lives of people living with dementia in long-term care facilities likely suppress the positive potential of boredom, as they are unable to respond to its prompts by engaging in alternative pursuits.

### Boredom in people living with dementia

Even though the three discussed signature markers of boredom (meaning, attentional ability and agency) obviously relate very well to dementia-specific research and the living environment of long-term care facilities, ‘we know almost nothing about boredom in older adults’ [33 p.32] and even less about boredom in people living with dementia [13]. Recently published systematic reviews on boredom in old age point to the scattered and sparse study landscape in this field of research [13, 33]. An et al. [13] focused on boredom during leisure time in older adults. The authors included eight studies, only one of which was conducted in residential long-term care and none of them examined people living with dementia [13]. With a broader scope, Ros Velasco [33] identified 49 publications, but only 26.5% of them were totally focused on boredom and older adults. Residential long-term care was examined in 19 studies, five of which focused specifically on people living with dementia. The synthesis of the setting specific literature revealed only fragments and examples of causes and consequences of boredom in residential long-term care, while the identified studies on people living with dementia refer mainly to the research of Cohen-Mansfield and colleagues. The studies highlight boredom in the context of unmet needs [9] and identify correlations between boredom and delusions/ hallucinations [34, 35]. However, both reviews conclude that boredom is a potential risk to successful ageing and has a negative impact on the health and quality of life of older people [13, 33].

In fact, there are hardly any specific studies on the impact of boredom on people living with dementia. In accordance with the presented research of Cohen-Mansfield and colleagues, the few existing studies confirm that boredom is associated with behavioral and psychological symptoms of dementia [36]. The associated behavioural and psychological symptoms vary greatly and range from apathy, depression and anxiety to very frequent physical aggression [37, 38]. However, the existing studies do not raise the question of when and why boredom is associated with which type of reaction [39]. Previous studies indicate that different causes of boredom are associated with different experiences and reactions [17]. It has been found that individuals experience five different types of boredom, which can be qualified according to their level of valence and arousal [14, 40]: indifferent (very low arousal, slightly positive valence), calibrating (low arousal, slightly negative valence), searching (medium arousal, medium negative valence), reactant (highest arousal, much negative valence), and apathetic (very low arousal, most negative valence) boredom. However, research into the types of boredom is still in its infancy and is mainly limited to the educational context. Nevertheless, the applicability of the identified types in other institutional settings, such as residential long-term care, is emphasised as a research desideratum [14, 40].

### Purpose of the study

The state of research reveals a fragmented and scarce body of evidence and points to the urgent need for more empirical research about the causes, consequences and experiences of boredom in old age, especially for the highly vulnerable group of institutionalized people living with dementia [13, 33, 41]. A better understanding of the everyday lives of people living with dementia in residential long-term care and the associated experience of boredom could help to identify potential opportunities for practical improvements of daily routines. Moreover, to learn more about different types of boredom would allow a precise tailoring of person-centered intervention approaches to prevent and reduce boredom in people living with dementia. In order to address the identified research gaps, this study pursues two objectives:

1. To explore the everyday life of people living with dementia in residential long-term care facilities with regard to (a) the type and frequency of activities during the day and (b) the lived experience of daily routines.
2. To explore the lived experience of boredom in people living with dementia in residential long-term care facilities with regard to (a) the experienced frequency of boredom and (b) the perceived type of boredom.

## Methods

### Study design

Using a merged methods approach [42] quantitative data is collected via informant-rated ecological momentary assessment and combined with qualitative interviews. The use of ecological momentary assessment provides an objective and extensive, real time picture of everyday activities of people living with dementia in residential long-term care, while interviews allow insights into the subjective experience of the daily routine from the perspective of people living with dementia [43]. This study is part of a larger research project on the daily lives and social health of people living with dementia in long-term care facilities in Germany (CaResource). The Ethics Committee of the Technical University Munich approved all methods and materials for data collection (47/20 S, 03 February 2020).

### Setting and sample

The data was collected in five residential long-term care facilities in the southern part of Germany (Bavaria). One facility is located in a rural area, four in urban areas. Between 62 and 208 residents live in the facilities, with the average number of residents being 136.0 ± 57.8. All facilities can be characterized as traditional care homes, where the primary focus is on quality of care and health outcomes, and everyday life is shaped by organizational routines and rules [44, 45]. The facilities did not offer alternative living arrangements, such as small-scale living, shared housing, green care approaches, or intergenerational living, nor were they part of dementia villages or group homes [44]. Additionally, there were no specific frameworks or strategies for involving informal caregivers, such as family members or friends. In four of the participating facilities, one ward each took part in the study; in one facility, two different wards took part. In each ward, a convenience sample of seven to ten participants were recruited for the momentary assessments by asking the care manager for residents fulfilling the following inclusion criteria: (1) dementia diagnosis noted in the care documentation, (2) mild to moderate level of dementia, (3) not cared for in bed, (4) living in the facility for at least two weeks. All residents who took part in the momentary assessments and had sufficient verbal communication skills were asked whether they would also like to take part in an interview about their daily routine. A total of 46 people living with dementia were observed, and 17 of these participants also took part in interviews about their daily routines and experiences of boredom. Table 1 presents the sample characteristics.

**Table 1:** Sample characteristics

Variable	EMA (*n* = 46)	Interviews (*n* = 17)
Age, years, mean ± SD	84.65 ± 7.15	85.29 ± 5.74
Gender, female (%)	89.13	88.24
Duration of stay, months, mean ± SD	28.04 ± 28.23	20.59 ± 18.82
Functional status, care level, mean ± SD	3.26 ± 0.88	2.94 ± 0.83
Cognitive status, DSS score, mean ± SD	6.50 ± 3.15	5.35 ± 2.30

Note. EMA = Ecological Momentary Assessment, SD = Standard Deviation, DSS = Dementia Screening Scale

All participants, as well as their guardians (in case of legal representation) provided written informed consent. Situational dissent concerning participation was respected at any time. All data was collected between October 2020 and June 2022. The survey phase was extended due to COVID-19 access restrictions in care facilities.

### Measures

#### Characteristics of the participants

The global functional status was determined by the care level of the German five-level system [46] (higher levels indicate a higher impairment of independence) which was taken from the participants’ care documentation. Cognitive functioning was assessed with the Dementia Screening Scale (DSS) [47]. The DSS is assessed from the perspective of the nursing staff in long-term care facilities and measures seven items on the domains of memory and orientation. The DSS total score varies between 0 and 14 (a higher score indicates more severe cognitive impairment). Internal consistency proved to be good, with a Cronbach’s alpha of 0.94 [47].

Age, gender and the duration of stay within the facility was taken from the participants’ care documentation.

#### Everyday activities

The German version of the Maastricht Electronic Daily Live Observation Tool (MEDLO-tool) was used to quantitatively analyse the type and frequency of activities throughout the day. In 2016, the original Dutch MEDLO-tool was developed with the aim to gain real time insight into the aspects of the daily lives of people living with dementia in residential long-term care facilities [48]. The MEDLO-tool demonstrated acceptable inter-rater reliability scores between 0.5 and 1 (Kappa or weighted Kappa scores) [48]. The MEDLO-tool and its detailed user manual was translated into German in 2018 [49].

The MEDLO-tool was applied in its entirety but only the observations of daily life activities were used for this merged methods study. The MEDLO-tool provides a list of 27 different activities to score. The activities reflect the everyday lives of people with dementia in long-term care facilities and include, for example, eating and drinking, (self) care activities, watching television, reading, and having a conversation. In addition to these specific activities, the three activities of sitting/lying down (the resident is sitting or lying down but is awake, there is no activity taking place), conscious resting (e.g. the resident is deliberately placed in bed by the care staff), and meaningless (repetitive) behavior (e.g. tapping on table, rubbing hands without reason, mumbling) can be scored. If the resident is not observable or performs an activity other than those listed, this can also be documented.

#### Daily routine and experience of boredom

A qualitative semi-structured interview was conducted to give people living with dementia the opportunity to share the experiences of their daily routine and the occurrence of boredom in it. To address challenges associated with interviewing people with dementia [50] specific recommendations were considered during data collection. The interview situation started with an introductory statement about the aim and procedure of the interview to provide orientation and to remind interviewees about the general focus of the interviews [51]. The interview guide consists of two main questions: (1) “What does your daily routine look like here?” and (2) “Do you experience boredom in your everyday life?“. Follow-up questions are asked to stimulate further narratives. These include, for example, questions about fixed activities in the course of the day or, based on the participants’ statements, what usually happens next in the course of the day. If participants report on experiencing boredom, they are asked how often boredom occurs in everyday life and whether they would like to describe how they experience it. The communication style and the complexity of follow-up questions were continuously tailored to the individual needs of each participant.

### Procedure and material

Observational data was collected on two consecutive days using the tablet application of the MEDLO-tool. All participants were observed from 7 am to 7 pm by previously trained research staff with experiences in the field of long-term care. Observations were made in 20-minute slots, within each participant was observed for one minute, after which the observer scored the activity. The order in which the participants were observed was randomly generated by the MEDLO software for each observation time point. The researchers observed the residents where they were at the time and documented the situation without influencing it (e.g. through interaction with the residents). A total of 2760 momentary assessments were made during the observation period.

The interviews were conducted in the same week in which the participants were observed. The research staff who carried out the observations also interviewed the people with dementia. Depending on the participants’ preferences, the interviews were conducted in their rooms or in a quiet place in the communal area. All interviews were digitally recorded and transcribed verbatim. The length of the interviews ranged between 3.5 and 36.5 min with an average length of 15.5 ± 9.5 min, resulting in a total corpus of 104 pages of transcripts. Passages in the interviews that did not relate to the interview topic were excluded from the material for the analysis.

### Analysis

Transcripts are analysed using qualitative content analysis based on Mayring [52]. The procedures of (a) summarizing, (b) scaling-structuring and (c) content-related structuring are applied to the material.

- Summarizing is used to inductively develop categories that reflect the structure of the daily routine reported in the interviews. Within these categories, summarizing is used again to synthesize the narratives.
- Scaling structuring is used to categorize the interviewees according to the frequency of boredom experienced. Frequency of experienced boredom is scaled into three deductive categories: (1) never (the feeling of boredom is not present in the person’s everyday life), (2) sometimes (the person experiences boredom but not as a prevailing condition in his or her everyday life), (3) always/often (the person experiences boredom as a constant/prevailing condition in his or her everyday life).
- Content-related structuring follows the five deductive categories of boredom types [40]: (1) indifferent boredom, (2) calibrating boredom, (3) searching boredom, (4) reactant boredom, and (5) apathetic boredom.

Any meaningful element, even if it only consists of a single word, is defined as a coding unit. The interview of one person forms the context unit; the entire material represents the unit of analysis. Each phrase that clearly refers to a specific interview question is defined as a selection criterion. The entire material was analysed by two coders. The category system and the coding agenda were steadily revised and complemented. A reliability proof of the categories was made, whereby disagreements between the two coders were resolved by discussion and consensus. Finally, parts of the qualitative data were quantified by calculating frequencies. MAXQDA 2020 was used for the qualitative data analysis.

The observation data was analyzed descriptively, and frequencies and distributions were calculated. Most frequent activities are displayed in a treemap; the distribution of the most frequent activities throughout the day is displayed in one-hour time slots in a trend diagram. Quantitative analysis was made using SPSS Statistics (Version 29.0.). Microsoft Excel was used to visualise qualitative and quantitative data (e.g. joint displays and treemaps).

## Results

### Everyday life and daily routine

**Fig. 1:**
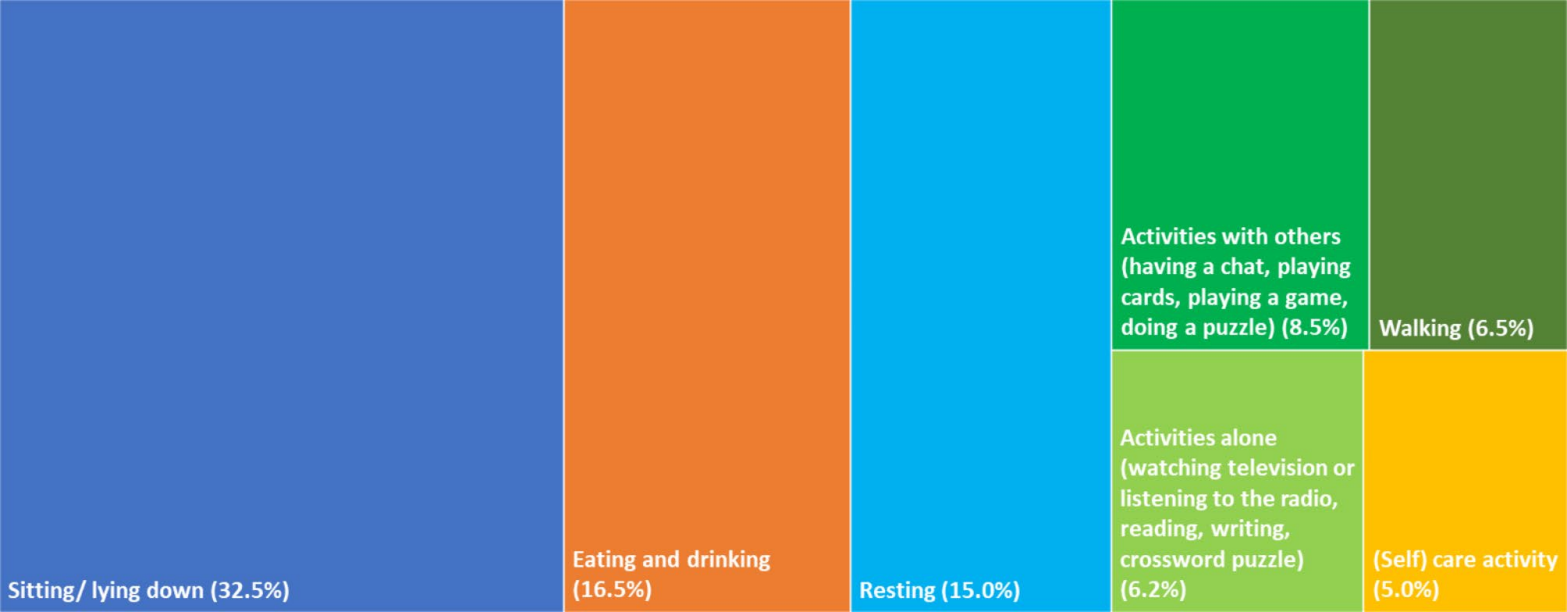
Most frequent everyday activities, n = 2734 . *Note*: All other activities were observed < 1% of the time, the sum of the observed proportion of these activities is 4.6% in total. In 5.2% of the time points, the activity was not observable

A total of 2724 momentary assessments and 17 interviews are included to describe the everyday life and the daily routine of people living with dementia in residential long-term care facilities. Of the 30 everyday activities listed in the MEDLO-tool, 23 were observed to occur. Figure 1 shows the nine activities that were observed at more than 1% of the time points. Leisure activities that are predominantly carried out alone and those that are predominantly spent together with other people were each grouped into one category. The most frequently performed activities are sitting/lying down, eating and drinking, and resting/sleeping. Summing up the categories sitting/lying down and resting/sleeping (colored blue in Fig. 1) shows that residents spend nearly the half of their time (47.5%) doing nothing at all. Participants spend just over 20% of their time on eating/drinking and care activities (colored orange in Fig. 1). The remaining third of the time is used for leisure activities and walks (colored green in Fig. 1).

**Fig. 2:**
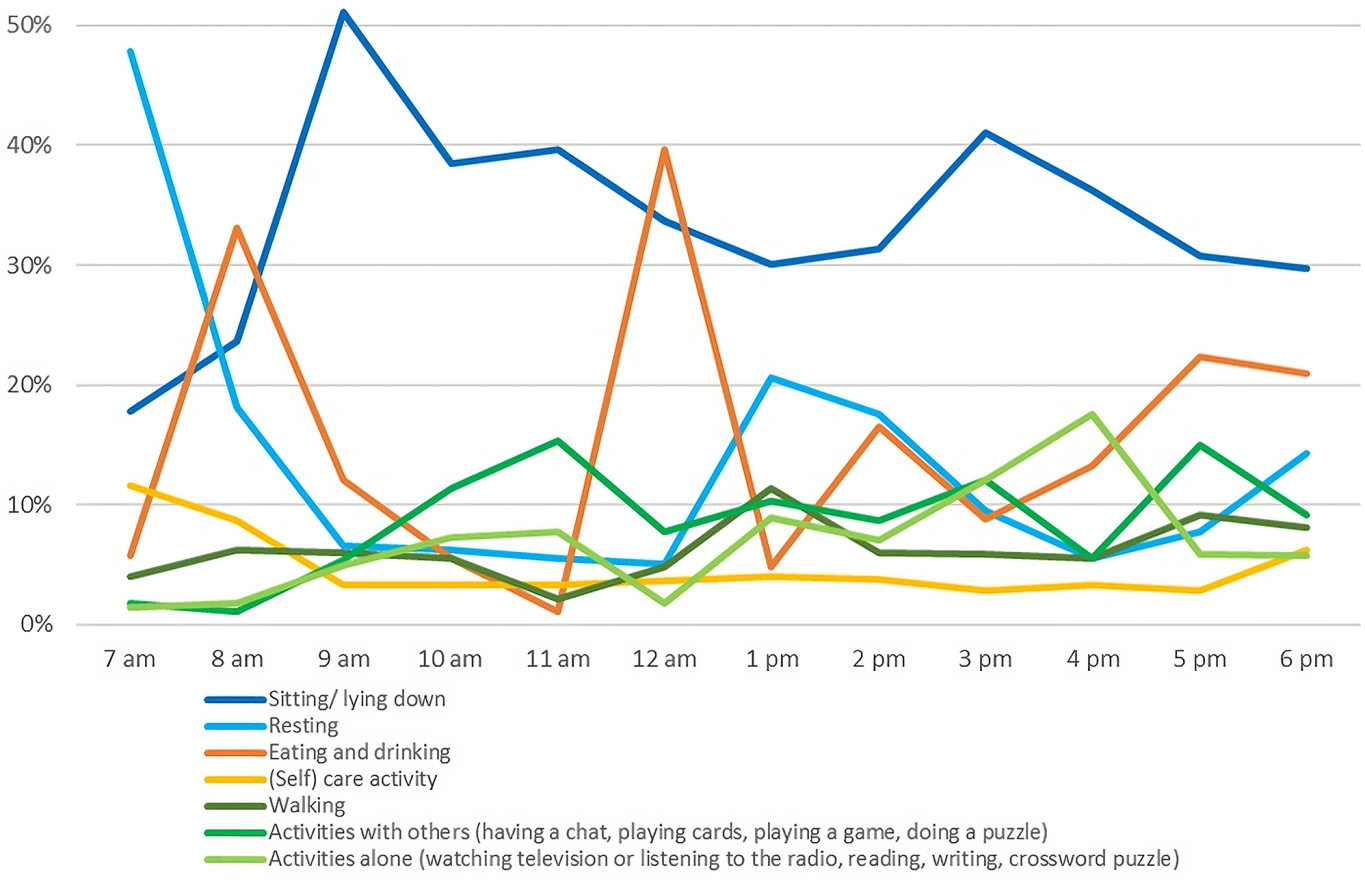
Everyday activities during the course of the day, n = 2466. *Note*: The 100% represent all observed activities. The percentages therefore refer to the proportion of observed activities at the respective time point

From the participants’ narratives, a five-part structure of the daily routine emerges, which is strongly determined by shared meals: (1) the morning lasts until after breakfast (approx. 9 am), (2) the late morning ends with lunch (approx. 12 noon), (3) the afternoon ends with dinner (approx. 5 pm), (4) dinner ends at 6 pm at the latest and then (5) the evening begins. Not all interviewees reported on the entire course of the day, thus the number of underlying interviews is given separately for each part of the day. Figure 2 shows the percentage of the most frequent everyday activities during the course of the day. In the section outlined below, the everyday life is described according to the identified structure of the daily routine, whereby the lived experiences resulting from the interviews are complemented and merged with the observations.

#### Morning

In the interviews, 13 people with dementia described how they spend their mornings when asked about their daily routine. The participants’ day starts individually, with a time slot from 6 to 9 am, with most participants reported that they get up between 7 and 8 am. Some people with dementia mentioned that they do not have a set time to get up or that they get up later here than they used to at home. Residents also reported that their getting up time depends on when the care staff get them out of bed to carry out morning care. Activities that are carried out before breakfast include reading, washing/showering and getting dressed (with and without the support of care staff), planning the day and going to the communal room earlier to wait for breakfast together and listen to music. For all residents, breakfast is the first fixed part of their daily routine, and all stated that they eat breakfast in the community. One resident describes the start of her day as follows:

*‘So you get up, then you get dressed or showered, depending, then you go to breakfast, wait there, listen to music, and wait until 8 o’clock until breakfast comes.’*

The narratives of the interviewees correspond very well with the activities observed in the morning. Figure 2 shows that the frequency of resting decreases between 7 and 9 am and eating/drinking peaks at 8 am. Before breakfast, mainly (self) care activities are carried out, the walking activities observed probably concern the way to and from the common room where breakfast is served.

#### Late morning

The descriptions of the seven people with dementia who reported on how they spend their late mornings vary. Reading, cleaning up, gathering information, writing or going out into the garden when the weather is nice are activities that were described. Only a few participants reported (unspecific) structured group activities. Some residents who have not washed/showered in the morning do so after breakfast. Some stay in the common room after breakfast and *‘have a look around’.* The observations in the late morning show that leisure activities are carried out with others more often than activities alone. One resident, for example, described the late morning as follows:

*‘After breakfast*,* at about nine*,* half past nine*,* you sit at the table and then you either go out into the garden or you do something*,* you go into your room or whatever everyone likes to do.’*

It is reported that the time until lunch ‘*passes quickly’*, but also that the late morning is characterised by *‘slowly passing time while waiting for lunch’* and ‘*idle time’*. One interviewee mentioned that *‘you can go to bed again straight after breakfast’*. Directly after breakfast is the peak of idleness in the observations, i.e. the time is spent without any particular activity, but awake (sitting or lying down).

#### Noon

The next fixed point in the daily routine is lunch, as stated by 11 people with dementia. In the residents’ narratives, lunch begins at different times, but it usually takes place between 11.30 and 12.30 am. Lunch also stands out in the observations as the main activity around 12 noon. Lunch is eaten in the community by all participants interviewed, as the following interview segment illustrates:

*‘Then it’s lunchtime, yes, and then we all sit out there and eat.’*

#### Afternoon

A total of 14 interviewees reported how they spend their afternoons. Immediately after lunch, most residents mentioned taking a nap or an afternoon rest. Some mentioned that coffee and cake are served in the afternoon, around 2.30 pm. Immediately after lunch is also the time when observations of the rest periods during the day reach their peak, followed by an increase in eating and drinking, which reflects the reported afternoon coffee. One of the interviewees reported that it depends on the staff on duty whether coffee and cake are offered. Two interviewees report that they sometimes have coffee with relatives in the afternoon. When the weather is nice, some residents go out into the garden to take a short walk with the staff, relatives or on their own, or to chat with other residents. Reading or watching television were each mentioned by one resident. Observations show that more activities are done alone in the afternoon than together with other residents, which contrasts with the late morning. However, after coffee sitting or lying down without any activity increases again and remains the predominant activity during the afternoon. The following quote gives an exemplary insight into the afternoon routine of one interviewed person:

*‘After lunch, that’s when you usually rest, I usually sit here in my chair and yes, I watch television or I go to bed, depending on how I feel and yes, that’s how you spend the afternoon’.*

#### Evening

Eight residents reported on their evening routine. The last fixed point in the reported daily routine is dinner. Dinner is at different times in the interviews, but between 5 and 6 pm in all cases, what is also reflected in the observations. Some residents described that the dinner is a good opportunity to socialise or that they spend some time in the common room after dinner to chat with the other residents. Most people watch TV after dinner, do some (self) care activities and go to bed between 7.30 and 8.30 pm. The observations also show a decrease in activities directly after dinner, with a slight increase of (self) care activities. One interviewee reported on her evening as follows:

*‘Yes, it’s quiet in there at 6 pm, or a quarter past 6 pm, everyone goes to their room then. Then I just go into my room and watch the evening programme. Then I wait again until the nurse comes and gets me ready for bed. Yes, and then it’s usually half past seven and she puts me to bed.’*

### Experienced boredom

#### Frequency of boredom

**Fig. 3:**
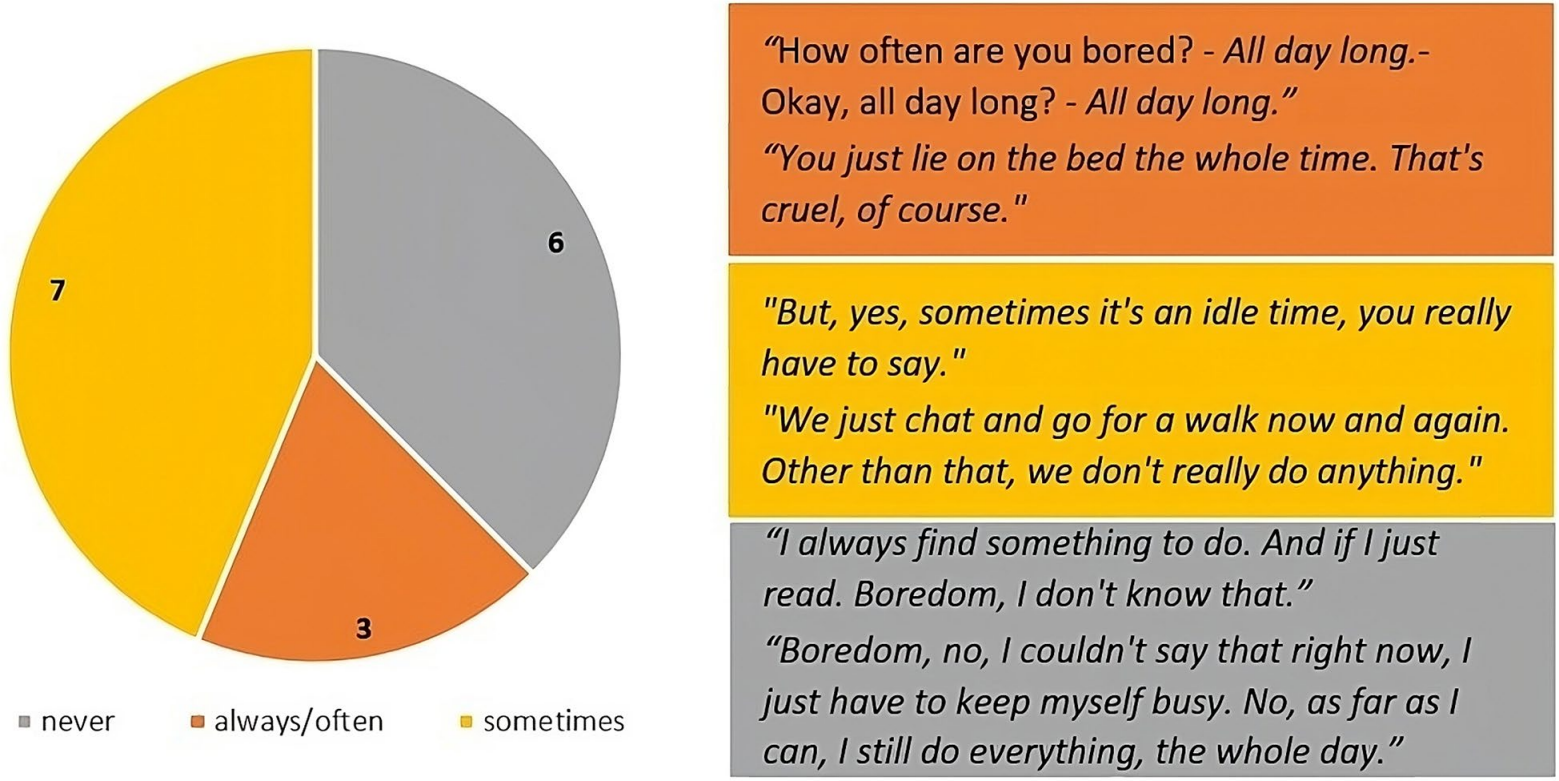
Frequency of boredom, n = 16. *Note*: One interviewee did not provide any information on this question

Of the 17 people with dementia who took part in the interviews, 16 gave insights into their experiences of boredom in their everyday lives. Figure 3 shows the frequency of boredom experienced and the corresponding quotes in a joint display. The largest proportion of interviewees (43.8%) mentioned that they experience boredom in their everyday lives, but do not perceive this as a constant/prevalent condition. Just under 20% (18.7%) of participants always/frequently feel bored, while more than a third (37.5%) never experience this feeling in their everyday lives.

#### Types of boredom

**Fig. 4:**
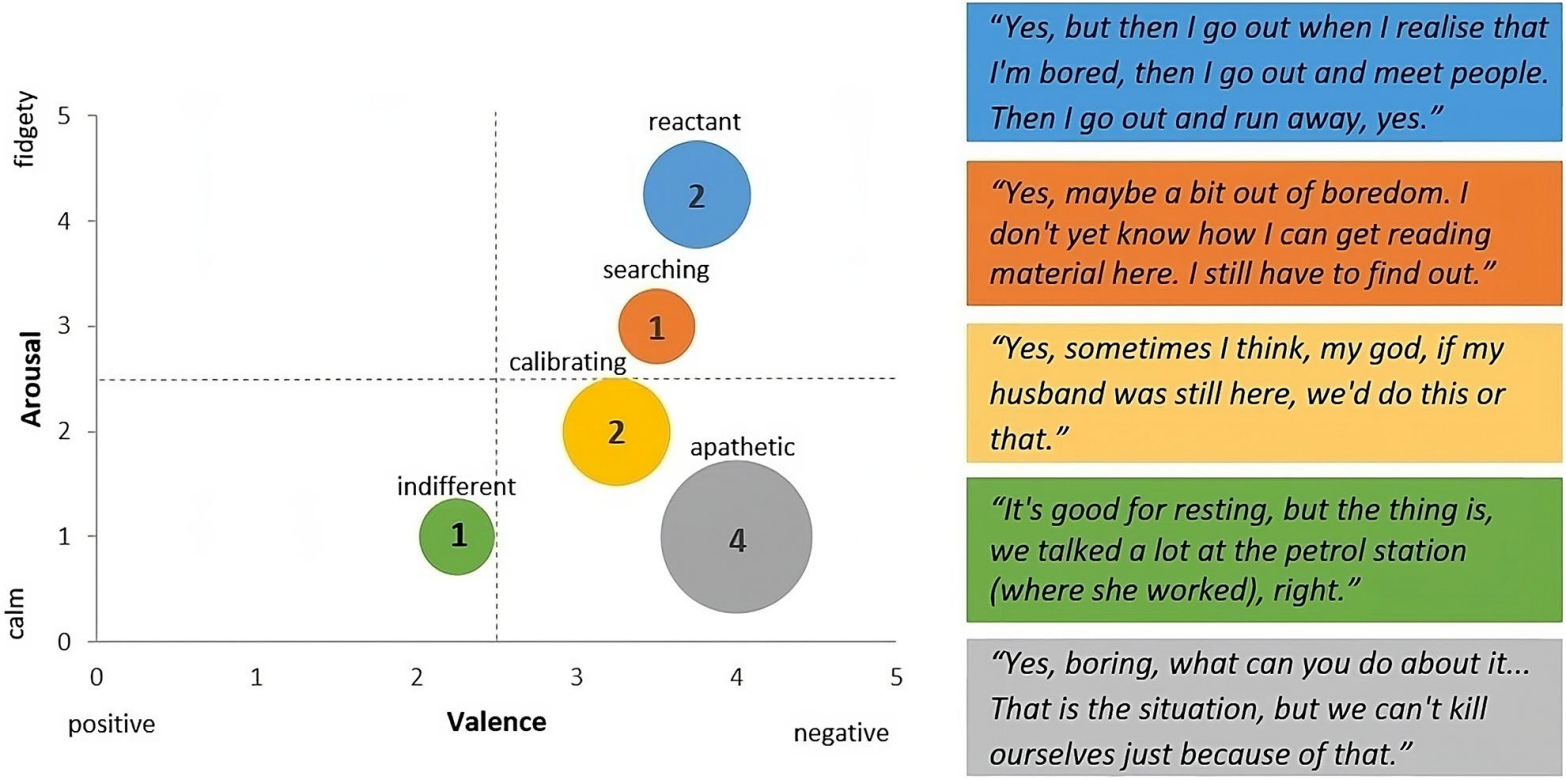
Types of boredom, n = 10. *Note*: the illustration of boredom types is inspired by Fig. 1 in the publication by Raffaelli et al. [14, p.2457]

The ten interviewees who reported experiencing boredom in their everyday lives were categorised according to the types of boredom [40]. Figure 4 shows the frequency of the boredom types with their position according to the dimensions of valence and arousal and the corresponding quotations in a joint display.

All five types of boredom could be identified in the participants’ experiences. The largest proportion of interviewees experiences predominantly apathetic boredom, which is characterised by the absence of both positive and negative affective states and comes very close to the feelings associated with learned helplessness and depression. The experience of reactant boredom characterises the interviews of two people with dementia. This type was associated with some kind of restlessness and the need to escape from the situation. Calibrating and searching boredom were found to be very similar boredom types. A distinctive attribute proved to be whether a participant only showed a receptivity to boredom-reducing options and had digressive thoughts that mostly related to the past (calibrating boredom) or whether a person actively searched for concrete alternative behaviours in the present (searching boredom). One interviewee reported that boredom can also be very relaxing and that she enjoys the peace and quiet (indifferent boredom).

## Discussion

By merging 2760 momentary assessments with 17 interviews, this study investigated the everyday lives and boredom experienced by people living with dementia in residential long-term care. The residents spend almost half of their day doing nothing and follow a routine that is strongly determined by communal meals. Over 60% are bored, with 18.7% describing boredom as a constant/prevalent state in their everyday lives. All five types of boredom are reflected in the interviews, with apathetic boredom being the most common.

### Everyday life and daily routine

Half a century later, the present results reinforce the central finding of Gottesman and Norman’s [4] study by showing that residents spend large parts of the day doing nothing. With respect to a more recent and comparable study [27], the present results show lower rates of inactivity during the day (48% vs. 57%), but a similar distribution of activities in the (late) morning (more often with others) and in the afternoon (more often alone). Focusing on activity rates in course of the day, Adlbrecht et al. [27] identified the highest rates in the morning, covering the entire period between 7 am and 12 am. The present results provide a more differentiated insight into this time of day, which only partially confirms this finding: The period between 7 am and 12 am shows high levels of activity (e.g. peaks in (self) care and eating and drinking), but also the daily peak of inactivity. This indicates that the identified five-part structure of the daily routine, which is rather small-scale and not based on fixed time periods, but on the daily structure depending on meals, may offer a more suitable time frame for the recording and interpretation of activity patterns in this context. However, the identified significance of eating and drinking as the most frequently observed activity and the determining factor for the daily routine is consistent with the findings of Stöhr et al. [26], who describe the daily routine in residential long-term care as ‘*after mealtime is before mealtime*’. Mealtimes not only influence the structure of the day, but they also emerged in the participants’ narratives as social anchors in the daily routine. This finding is consistent with previous research on social interaction among people with dementia in residential long-term care, which shows that social contact with peer residents is more likely to occur during shared meals than through structured group activities [53, 54]. As the interviews revealed, dinner in particular seems to be perceived as a good opportunity to socialise with peer residents. This perception is in line with an observational study that identified the daily peak of social interaction between residents during the evening meal [10].

By presenting the observed activities in one-hour time intervals, this study is able to determine for the first time the parts of the day with the highest proportion of time without any activity, namely directly after breakfast and between afternoon coffee and dinner. Interestingly, apart from a few sporadic glimpses of the experience of having nothing to do after breakfast, phases of idleness are hardly reflected in the interviews on the daily routine. The synthesis of the reported daily routines suggests that the interviewees have a full schedule without major time gaps. There are some possible explanations for the divergence between the objectively existing inactivity and the almost non-existent periods of idleness in the subjective descriptions of the daily routine. One reason could be that people with dementia are aware of periods of idleness during their day, but are ashamed to talk about it because they do not want to be perceived as being lazy or unable to keep themselves occupied [55]. A second explanation could be that the observed idle time is not perceived as such. Beerens et al. [56] have shown that residents with dementia can be engaged even if they are not actively performing an activity but are actively looking around. In line with this, Nygaard et al. [12] reported that residents with dementia sometimes consciously prefer the role of observer without wanting to actively participate in activities. In addition, Tierney et al. [20] emphasise the value of passive participation in activities: Watching activities or simply being with others can be perceived as meaningful by people with dementia.

In the few examples in which the participants in this study reported time without a specific activity, it was usually in the context of waiting for something, particularly for the next meal. In accordance with this, Harper Ice [5] described waiting times around meals as a very common and typical phenomenon in everyday life in residential long-term care – particularly in relation to immobile residents who need to be transported to and from the dining room. Waiting for things to happen was identified as common experience in people with dementia in a meta-synthesis of the experience of lived time [57]. On closer inspection, the waiting time can be associated with both positive and negative aspects, which are reflected in the results presented. On one hand, passing time together while waiting was found to facilitate the development of relationships with peer residents [54]. On the other hand, the perception that time passes too slowly while waiting is associated with the experience of boredom, as a slower perception of time is considered a correlate of boredom [14].

However, focusing on another correlate of boredom, lack of agency, which was assumed to be a typical feature of everyday life in residential long-term care, the results are rather unexpected. Neither the image of an ordinary day in a care home, characterised by a fixed structure full of predetermined activities [25], nor the narrative that residents perceive the daily routine as restrictive [12, 58], can be fully confirmed by the present results. Firstly, the results show a considerable degree of flexibility in the daily routine, as there are hardly any activities or times determined by the facility apart from the meals. However, flexibility is not always related to greater autonomy in the analysed interviews. Flexibility is associated with the residents’ freedom of choice, e.g. how they spend their time between meals, but also with the arbitrariness in the organisation of the daily routine by the staff. For example, the time of getting up or the offer of coffee and cake in the afternoon is described as dependent on the staff on duty and therefore neither influenceable nor predictable. This finding is consistent with the study from Førsund et al. [24], who also emphasise dependence on staff as an aspect that limits autonomy in the context of everyday activities in care homes. Secondly, although the interview guide used did not directly ask participants how they feel about their daily routine, it is worth noting that none of the residents expressed that they experience it as restrictive. In addition to the absence of a strict daily schedule, this may be related to a positive perception of the daily repetition of the same rhythm, as it gives people with dementia a sense of security [25]. However, the findings on the experience of boredom provide more specific insights into how everyday life in residential long-term care is perceived by people with dementia.

### Experienced boredom

The present results indicate that the problem of boredom in residential long-term care is somehow both overestimated and underestimated. Not everyone is ‘bored to death’; more than a third (37.5%) of the participants state that they are not bored. Surprisingly, the result for the prevalence of boredom corresponds almost exactly to those found in a US study of a representative sample of adults (63%) [59]. The presumption that care home residents (with dementia) are particularly affected by boredom is therefore called into question. Nevertheless, when we focus on those actually affected, boredom is most frequently experienced as an intensely negative feeling that is associated with hopelessness and frustration. Thus, it can be assumed that the significant consequences of boredom on quality of life and health [60] have so far been underestimated in this target group.

Following the pioneering study by Goetz et al. [40] and subsequent studies confirming the authors’ findings [61], this study is the first to reveal the existence of different types of boredom in people with dementia. In addition, the applicability of Goetz et al.‘s boredom typology was demonstrated for this target group, and interestingly, despite the small sample size of ten people, all five types could be identified. Due to the qualitative approach in this study, it was not arousal and valence values that were decisive for categorising the types, but rather the statements, thoughts and feelings associated with the respective boredom type [40]. Based on this categorisation process, the present study can add at least four target group-specific aspects to the set of type-specific statements, thoughts and feelings. (1) Digressive thoughts related to the calibrating boredom type were mostly concerned with the past and past possibilities for action. Turning to the past when experiencing boredom was also observed by van Tilburg et al. [62] in a sample of young adults. Tilburg et al.’s findings indicate that boredom facilitates the recall of nostalgic memories, which in turn convey a sense of meaningfulness and thus mitigate the negative effects of boredom in the present. The boredom reduction function of reminiscence was also confirmed for people with dementia in previous research [63]. (2) With regard to the indifferent boredom type, relaxation and cheerful fatigue were reported against the background of a work-filled life so that boredom is now experienced as a pleasant contrast. (3) Similar to the description of Goetz et al. [40], statements reflecting reactant boredom indicated a restlessness and a strong need to escape the situation. However, in Goetz et al.’s investigation, the students were unable to leave the situation (lectures). In contrast, the interviewees reported that whenever they have these negative feelings and restlessness, they put their strong desire to leave the boredom-inducing situation into action. This finding demonstrates that people living with dementia can access the positive potential of boredom by responding to its implicit cue to ‘do something else!’ [31]. In this context, the interviews show that boredom motivated participants to seek social interaction, which represents a significant benefit. (4) The different situations to which boredom refers (boredom in the temporary situation of a lecture vs. boredom in everyday life) can certainly also explain the even more negative characteristics of the apathetic boredom type revealed in the interviews. The interviewees expressed high degrees of hopelessness and frustration, deep disappointment and silent resignation. In line with these findings, one of the few existing studies on boredom in care homes [64] indicates that residents lose their ability to think positively in the face of boredom. Furthermore, the authors found that residents attribute the cause of their disengagement to others, which is closely linked to the feelings of disappointment identified in the present interviews. The external attribution of the cause of the aversive state can already be found in the definition of boredom by Eastwood et al. [15] and therefore does not only seem to be valid for this target group. However, studies found that both environmental characteristics and a person’s propensity to boredom contribute to boredom [65]. As the disease progresses, people with dementia become increasingly dependent on their environment and thus also on the influence of environmental characteristics on the development of boredom.

### Implications

The present results indicate that although people living with dementia in residential long-term care follow almost the same daily routine, they perceive their everyday lives very differently: The spectrum of experiences ranges from a feeling of constant busyness to resigned boredom. This suggests that strategies aimed at avoiding and reducing the highly individualised experience of boredom are most effective when they adopt a personalised approach. Person-centered care provides a valuable intervention strategy to meet this requirement [66]. This philosophy takes a holistic view of people living with dementia and places the unique needs, values, preferences and subjective experiences of the individual at the centre of its actions [66]. Providing individualised activities, meaningful engagement and enabling a purposeful life is an important pillar of this approach [66, 67]. Person-centred dementia care encompasses a wide range of interventions, including common non-pharmacological measures such as physical activities and cognitive training, but also measures that focus on adapting the environment and organising care [67, 68]. Currently, the body of evidence on person-centred dementia care includes neither specific interventions against boredom nor boredom as a relevant study outcome [67–69]. Consequently, integrating the topic of boredom in practice and research of person-centred dementia care may be proposed as a future field of development. This would also include initiatives to increase carers’ knowledge and awareness of the causes, experiences and consequences of boredom in people with dementia in order to recognise this aversive state in everyday life and react accordingly [13]. Further, designing specific interventions to address boredom can draw from existing knowledge about health-promoting, meaningful activities. Evidence-based approaches, including individualized recreational activities, reminiscence therapy, music therapy, and multi-sensory stimulation, have demonstrated effectiveness in alleviating behavioral and psychological symptoms and enhancing the quality of life for people living with dementia in residential long-term care [70, 71].

Even though person-centred dementia care offers an ideal approach to making everyday life and activities in residential long-term care less boring, its implementation in practice is associated with a number of challenges, above all the limited time resources of staff [69]. Paddock et al. [58] found that staff in residential long-term care have the desire to support residents’ identities and individuality through personalised activities, but fail to implement them in everyday life, usually resulting in the provision of ‘one-size-fits-all’ activities. In order to avoid placing the entire time burden of tackling boredom on the shoulders of the staff, meaningful relationships with peer residents [10] and personalized interactive multimedia systems [72] can serve as valuable complements to person-centred dementia care in order to address boredom. Furthermore, evidence suggests that even non-facilitated meaningful activities, such as listening to music, using companion robots, or interacting with lifelike dolls, can enhance emotional well-being, reduce agitation, increase happiness, and promote engagement among people living with dementia in long-term care [73]. These activities also offer a promising way to combat boredom with minimal demands on nursing staff time.

Even though this study offers initial insights into the world of boredom among people with dementia in residential long-term care, many gaps remain that need to be filled by future research. Three of these, which can build on the present results, should be highlighted: Firstly, future studies should measure the prevalence of boredom in residential long-term care in larger representative samples in order to better estimate the extent of the phenomenon. Second, to gain a deeper understanding of the factors that determine the occurrence and type of boredom in this target group, the influencing personal and environmental factors need to be explored. Thirdly, future studies should investigate the experience of boredom in specific recurring situations in the daily lives of people with dementia in residential long-term care, for example through situational interviews.

### Strengths and limitations

The greatest strength of the current study is its merged methods approach. By merging objective, real-time data from the momentary assessments with the lived experience of the daily routine of people with dementia, a rich and nuanced picture of daily life in residential long-term care was created. Furthermore, this is the first study to analyze the frequency and type of boredom in people with dementia, which may contribute to the future development of tailored and person-centred interventions to reduce boredom in this target group. However, the present study also has some relevant limitations. The data collection took place during and after the COVID-19 pandemic, although always at times when the restrictions on social contacts were very relaxed or not applied, but this could have influenced the results. The observation days were only carried out on weekdays; the inclusion of weekend days would certainly provide more insight into everyday life as a whole. The generalizability of the results is limited by the exclusion of people living with severe dementia. Obtaining the views and perspectives of individuals in advanced stages of the disease on their daily routines and experiences of boredom requires alternative methods, as verbal communication is often impaired at this stage [74]. Future research should focus on exploring the everyday life of people living with severe dementia using assessment approaches that enable them to express their thoughts and feelings, such as talking mats, flexi-boards for augmentative and alternative communication (AAC), or preference-sorting templates [75]. Furthermore, results on boredom prevalence relies on a single-question and we did not provide any additional explanations of the term boredom during the interviews. This may have led to the participants defining boredom in different ways and therefore the answers are limited in their comparability. The categorisation of boredom types was not based on quantitative data on arousal and valence, but the interviews were coded according to their similarity to the statements, thoughts and feelings characterising the respective boredom type. For this reason, and because of the small number of people typified, the present study can only be considered an initial exploratory pilot study on the types of boredom experienced by people with dementia.

## Conclusions

By merging objective, real-time data from 2724 momentary assessments with the everyday experiences of people living with dementia, this study provides a unique and nuanced picture of daily life in residential long-term care. Notably, it is the first study to analyze the frequency and types of boredom among this population, applying Goetz et al.‘s typology to demonstrate the existence of distinct boredom categories. The findings reveal a striking divergence: while daily routines are often characterized by inactivity and structured primarily around shared meals, the subjective experiences of boredom range widely. These include pleasant relaxation in some cases, but also profoundly negative emotions such as hopelessness and frustration. This underscores the complex, highly individual nature of boredom in dementia care. These results suggest that interventions targeting boredom must be highly personalized to address the individual needs and emotional states of residents. Person-centred dementia care, which emphasizes tailoring activities and environments to each individual’s preferences and abilities, offers a promising approach. Integrating boredom management strategies into both practice and research in person-centred care could enhance residents’ quality of life and reduce negative emotional outcomes.

## Data Availability

Datasets generated and analyzed during the current study are available from the first author upon reasonable request.

## Abbreviations

EMA: Ecological momentary assessment
DSS: Dementia screening scale
SD: Standard deviation
